# Characterization of the High-Quality Genome Sequence and Virulence Factors of *Fusarium oxysporum* f. sp. *vasinfectum* Race 7

**DOI:** 10.3390/jof10040242

**Published:** 2024-03-23

**Authors:** Dingyi Yang, Xiaojun Zhang, Yuqing Ming, Chenglin Liu, Xianlong Zhang, Shiming Liu, Longfu Zhu

**Affiliations:** 1 National Key Laboratory of Crop Genetic Improvement, Huazhong Agricultural University, Wuhan 430070, China; yangdingyi@webmail.hzau.edu.cn (D.Y.); xjzhang@webmail.hzau.edu.cn (X.Z.); mingyq@webmail.hzau.edu.cn (Y.M.); liuchenglin@webmail.hzau.edu.cn (C.L.); xlzhang@mail.hzau.edu.cn (X.Z.); 2 Hubei Hongshan Laboratory, Huazhong Agricultural University, Wuhan 430070, China

**Keywords:** *Fusarium oxysporum* f. sp. *Vasinfectum* race 7, *Fov*, comparative genomics, Fusarium wilt, secreted in xylem, cotton

## Abstract

*Fusarium oxysporum* f. sp. *vasinfectum* (*Fov*) is a common soilborne fungal pathogen that causes Fusarium wilt (FW) disease in cotton. Although considerable progress has been made in cotton disease-resistance breeding against FW in China, and the *R* gene conferring resistance to *Fov* race 7 (FOV) in Upland cotton (*Gossypium hirsutum*) has been identified, knowledge regarding the evolution of fungal pathogenicity and virulence factors in *Fov* remains limited. In this study, we present a reference-scale genome assembly and annotation for FOV7, created through the integration of single-molecule real-time sequencing (PacBio) and high-throughput chromosome conformation capture (Hi-C) techniques. Comparative genomics analysis revealed the presence of six supernumerary scaffolds specific to FOV7. The genes or sequences within this region can potentially serve as reliable diagnostic markers for distinguishing *Fov* race 7. Furthermore, we conducted an analysis of the xylem sap proteome of FOV7-infected cotton plants, leading to the identification of 19 proteins that are secreted in xylem (*Fov*SIX). Through a pathogenicity test involving knockout mutants, we demonstrated that *FovSIX16* is crucial for the full virulence of FOV7. Overall, this study sheds light on the underlying mechanisms of *Fov*’s pathogenicity and provides valuable insights into potential management strategies for controlling FW.

## 1. Introduction

The *Fusarium oxysporum* species complex (FOSC) comprises a diverse group of fungal pathogens capable of infecting over 100 plant species worldwide [1]. It comprises a diverse collection of host species’ distinct forms, known as *formae speciales* (abbreviated: f. sp.), that exhibit host-specific pathogenicity. These *formae speciales* are specialized to infect particular plant hosts, resulting in destructive vascular wilt diseases in economically important crops such as cotton, tomato, banana, and melon, thus leading to tremendous economic losses [2].

*Fusarium oxysporum* f. sp. *vasinfectum* (*Fov*), a member of the FOSC, is responsible for causing Fusarium wilt (FW), a tremendously damaging disease that affects cotton (*Gossypium* spp.) worldwide [3,4], The chlamydospores can survive in the soil for several years or even decades in the absence of a host, making the control and eradication of field wilt disease challenging [4,5]. Infected plants display symptoms such as stunted growth, leaf wilting and chlorosis, defoliation, vascular discoloration, and ultimately plant death [6]. Based on the specificity of limited DNA sequences and variations in pathogenicity toward diverse plant species, the *Fov* isolates are currently subdivided into eight pathogenic races (races 1–8) [6]. Phylogenetic analyses using housekeeping genes and mitochondrial small subunit (mtSSU) rDNA have further categorized these isolates into four lineages. Lineage I consists of race 3 and 5, lineage II consists of races 1, 2, and 6, lineage III consists of race 8, and lineage IV consists of races 4 and 7 [7]. Among them, race 1 and race 2 belong to the nematode-dependent pathotype, as they require the presence of the root knot nematode (*Meloidogyne incognita*) for disease development [5]. Race 4 was initially discovered in India and has become the predominant race affecting cotton production in California [8,9,10]. Races 7 and 8 were first described in China in 1985 [11], and races 3, 7, and 8 are the most commonly encountered races in the country, with race 7 being the most widespread and exhibiting the highest degree of virulence [12]. Race 7 is currently genetically indistinguishable from race 4 based on DNA markers and limited gene sequences, and both races belong to the same vegetative compatibility group (VCG) of *Fov* [13,14]. However, they can be differentiated based on their varying pathogenicity on different hosts [6,7].

The most extensively studied member of the FOSC is *F. oxysporum* f. sp. *lycopersici* (*Fol*), the causal agent of tomato wilt [1]. During infection, *Fol* secretes a multitude of pathogenicity-related proteins into the xylem sap of tomato plants, effectively suppressing the plant’s immune response. These secreted proteins are collectively referred to as secreted in xylem (SIX) proteins [15,16,17]. Within *Fol*, a total of 14 distinct SIX proteins have been characterized, with SIX4, SIX3, and SIX1 specifically identified as the effector proteins Avr1, Avr2, and Avr3, respectively [16,18,19]. The completion of genome sequencing in the FOSC has significantly advanced our understanding of the molecular basis underlying pathogenicity [20]. Comparative genome analysis has revealed that the genomes of *F. oxysporum* strains are divided into two compartments: conserved ‘core’ chromosomes and lineage-specific (LS) chromosomes [20,21]. The LS chromosomes have been found to be essential for pathogenicity and can be horizontally transferred to non-pathogenic strains during co-cultivation, resulting in the acquisition of pathogenicity by recipient strains, indicating the potential role of LS chromosomes in host specificity [20,22]. Furthermore, it has been observed that most of the SIX proteins are located on the LS chromosome [17], further supporting the notion that the host specialization of *F. oxysporum* depends on the lineage-specific chromosomes [20,22]. However, our understanding of the evolution of *Fov* virulence for cotton and *Fov* effector genes is currently limited. Recently, scientists have successfully sequenced the genomes of different pathogenic *Fov* races, including races 1, 4, 5, 7, and 8 [20,23,24,25,26,27]. These studies have provided valuable resources for comparative genomic analyses, enabling the identification of genes or genomic features associated with cotton pathogenicity and facilitating the development of reliable and universal diagnostic tools. However, long-read sequencing for FOV7 has not been conducted, and our knowledge of *Fov* effector genes remains limited.

In this study, we utilized PacBio and Hi-C assisted assembly techniques to obtain an almost chromosome-level genome for FOV7. Through comparative genomic analysis, we identified the core region and lineage-specific region of FOV7. Furthermore, we analyzed the evolution of different races based on orthologous genes. Additionally, we collected xylem sap from cotton plants infected with *Fov* and performed mass spectrometry, which enabled the identification of 19 *Fov*SIX proteins in FOV7. Notably, we discovered that *Fov*SIX16 is essential for the pathogenicity of FOV7.

## 2. Materials and Methods

### 2.1. DNA Extraction and Genome Sequencing

*Fov* race 7 isolate F17 was cultured on potato dextrose agar medium (PDA) plates at 25 °C for three days. Subsequently, small fragments of the isolate were transferred into potato lactose broth (PLB, 200 g potato and 20 g lactose per 1 L) and incubated for four days. The broth was then filtered through four layers of sterilized cheesecloth, followed by centrifugation to collect the spores. The spores were ground thoroughly with liquid nitrogen. DNA extraction was performed using the DP305 plant genomic DNA extraction kit (TianGen Biotech, Beijing, China) by substituting the lysis buffer with a fungal-specific lysis buffer, while the remaining steps were identical to those used for plant DNA extraction. 

Library preparation and genome sequencing were conducted at Novogene (Tianjin, China) using PacBio Sequel sequencing platforms (PacBio Sequel, PacBio Biosciences, Menlo Park, CA, USA) and Illumina sequencing platforms (NovaSeq 6000, Illumina, San Diego, CA, USA). The second-generation short-fragment library had a size of 350 bp and underwent paired-end sequencing. The PacBio SMRT library had a size of 30 K and underwent single-molecule sequencing.

### 2.2. Genome Assembly and Gene Prediction

The high-quality PacBio subreads were de novo assembled using the MECAT2 software (version 20192026) [28]. The consensus sequences of the assemblies were subsequently polished in two rounds with the PacBio subreads using the pbmm2 and gcpp algorithms implemented in the SMRTLink software (version 10.2). Additionally, the assembled contigs underwent two additional rounds of polishing using the pilon software (version 1.23) [29], with approximately 50× Illumina paired-end reads. 

Prior to gene prediction, repetitive sequences in the genome of FOV7 were identified and masked. RepeatModeler was employed for the de novo identification and modeling of repetitive families. Subsequently, RepeatMasker was used to analyze and mask the identified repetitive elements [30]. Gene predictions were carried out for repeat-masked genomes using the Funannotate pipeline (version 1.8.8) available at https://funannotate.readthedocs.io/en/latest/ (accessed on 24 December 2021), which integrates ab initio gene prediction, homology-based prediction utilizing homologous genes from related species, and transcriptomic data sets. 

### 2.3. Genome Assembly Using Hi-C

Hi-C libraries’ construction and sequencing for FOV7 were conducted at SMART BIOTECH (Tianjin, China). Fresh spores of FOV7 were harvested to create Hic libraries. Initially, the samples underwent formaldehyde fixation, DpnII restriction enzyme digestion, end repair, and biotinylation of DNA ends. Subsequently, T4 DNA ligase was utilized to join interacting fragments. Following ligation, proteinase K was employed for de-crosslinking, and protein-bound DNA fragments were digested to isolate purified DNA. The purified DNA fragments were then fragmented into sizes between 300 and 500 bp, and biotinylated DNA fragments were separated using Dynabeads^®^ M-280 Streptavidin (Life Technologies, Carlsbad, CA, USA). Library sequencing was conducted on the Illumina NovaSeq6000 platform in PE150 sequencing mode. About 100 × depth data were generated and these clean data were used to scaffold the genome by using ALLHiC software (version 0.9.8) [31]. 

### 2.4. Evolutionary Analysis

Orthologous groups among Fusarium species were identified using OrthoFinder v2.3.8 [32], followed by the application of the STAG method to deduce a phylogenetic tree encompassing these species [33]. *Fov* isolates used for evolutionary analysis are listed in Table S1.

### 2.5. Identification of LS Genomic Regions and Comparative Genomic Analysis

Genome alignment between FOV7 and *Fol* 4287 was performed using MUMmer (version 3.23) software [34] with parameter settings -maxmatch -c 90 -l 40. The alignments were filtered by running the delta-filter with the parameter -1 -i 90 -l 1000. The visualization of synteny between FOV7 and *Fol* 4287 was conducted using Circos (version 0.69) [35]. Identification of syntenic gene blocks was performed by using the python version of MCScanX software (jcvi 1.0.6) [36]. Synteny multicollinearity analysis between different *Fov* races was conducted using the NGenomeSyn tool (version 1.41) [37]. Genome alignment was performed by invoking the minimap2 software (version 2.17) [38], with the parameter setting -MinAlgLen 20000 to exclude alignment lengths shorter than 20 kb. *Fov* isolates used for comparative genomic analysis are listed in Table S1.

### 2.6. Identification of Secreted in Xylem (SIX) Protein of FOV7

Xinluzao 7, a susceptible cotton variety, was chosen for both the inoculation and control treatments to collect xylem sap. Prior to FOV7 inoculation, Xinluzao 7 was cultivated using hydroponics in a growth chamber. One week later, FOV7 inoculation was performed through root dipping. Roots of the prepared cotton plants were immersed into the spore suspension for 30 min before being transplanted into nutrient soil. The inoculation concentration was set at 1 × 10^7^ spores/mL, and a non-inoculated control was included. After 10 days of inoculation, xylem sap was collected when cotton leaves first exhibited wilt symptoms. The xylem sap collection method was performed as described previously with slight modifications [39]. The cotyledonary node of cotton seedlings was de-capitated using a sterile surgical blade. The initial two drops of sap were discarded to prevent contamination from the cytoplasm of cut cells and the sap was directly released during cortex cutting. A plastic tubing of approximately 5 cm in length was connected to the incision on the hypocotyl, and at hourly intervals, the sap was collected using a syringe into prechilled centrifuge tubes placed on ice. Sampling was carried out for 12 h, and the collected samples were stored at −80 °C. Each treatment was independently repeated three times, resulting in a total of six xylem sap samples. Label-free quantitative LC/MS proteomics analysis of the xylem sap samples was conducted at Novaseq (Tianjin, China). The modified filter-aided sample preparation (FASP) method was employed for extraction. After enzymatic digestion, desalination, and mass spectrometry, raw data were obtained. The resulting raw files were imported into Proteome Discoverer software (version 2.2) for database searching, peptide spectral matching, and protein quantification.

### 2.7. Fungal Transformation

*Fov*Δ*SIX6* and *Fov*Δ*SIX16* mutants were generated using a homologous recombination method, in which the coding sequence was replaced with a hygromycin resistance cassette. The plasmid was constructed by amplifying and fusing the upstream and downstream genomic sequences of *FovSIX16* into the pGKO-HPT vector, respectively. Related primers are listed in Table S2. Both fused plasmids were transformed into the *A. tumefaciens* strain GV3101 by electroporation. The *A. tumefaciens*-mediated transformation method was performed to generate the *Fov*Δ*SIX16* knockout mutants, following a previously described protocol [40].

### 2.8. Fov Inoculation and Infection Assays

These assays were performed as described previously [41]. Briefly, cotton seedlings were cultivated using hydroponics in a growth chamber before *Fov* inoculation. *Fov* inoculation was performed with a concentration of 1 × 10^7^ conidia per mL, and the cotton seedlings were subsequently transplanted into nutrient soil. The infection assay included disease index (DI) statistics, fungal biomass measure, and fungal recovery from infected cotton. The evaluation of DI adhered to the technical criteria outlined in GB/T 22101.4-2009 [42]. Quantification of fungal biomass was conducted by using qRT-PCR, estimating the relative quantity of Fov-specific DNA against cotton-specific DNA (UB7). For the fungal recovery assay, the first internode of infected cotton seedlings was excised, surface sterilized, cut into small sections, and then incubated on PDA solid medium at 25 °C.

### 2.9. Transient Expression in N. benthamiana

The complete coding sequence of *FovSIX16* and its signal peptide-truncated form (*Fov*Δ*spSIX16*) were amplified and subcloned into the pMDC84 vector using Gateway cloning (Invitrogen, Carlsbad, USA). Related primers are listed in Table S2. The resulting fusion constructs were then transformed into the *A. tumefaciens* strain GV3101 through electroporation. For the subcellular localization study, the Agrobacterium cell suspension containing *FovSIX16* and *Fov*Δ*spSIX16* was infiltrated into *N. benthamiana* leaves at an OD_600_ of 0.8. Additionally, the plasma membrane marker CBL and the nuclear marker HY5 were co-infiltrated with *FovSIX16* or *Fov*Δ*spSIX16* at an OD_600_ of 0.1. Sixty hours after infiltration, subcellular localization was observed using confocal microscopy (Leica, SP8, Wetzlar, Germany).

### 2.10. Yeast Signal-Sequence Trap System

The signal peptide sequences of *FovSIX16* were predicted using signalP software (version 5.0), and the predicted sequences were synthesized and subcloned into the pSUC2 vector. To serve as a positive control, the pSUC2-SP::Avr1b vector containing the signal peptide of Avr1b, a *P. sojae* RxLR effector, was used, while the pSUC2 empty vector served as the negative control. These plasmids were transformed into the YTK12 yeast strain. Signal peptide evaluation was performed using methods previously described in the literature.

## 3. Results

### 3.1. Genome Assembly and Annotation of Fusarium oxysporum f. sp. vasinfectum Race 7

**Figure 1:**
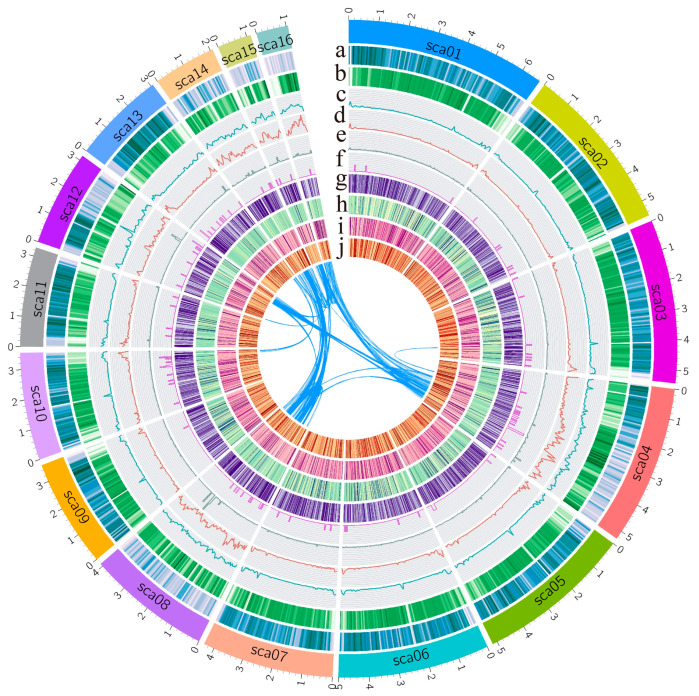
Genome features of *Fusarium oxysporum* f. sp. *vasinfectum* race 7 (FOV7). (a) Gene density in each scaffold. (b) GC contents. (c) Density of transposable elements (TEs). (d) Density of DNA transposons. (e) SNP density between *Fov* race 7 and race 4. (f) Indel density between *Fov* race 7 and race 4. All of these data are shown in 50 Kb windows. (g–j) Expression level of genes in vitro and in vivo in FOV7. Heatmap showing the expression level of genes in FOV7 cultures grown in Czapek Dox Broth Medium (g), as well as in FOV7-infected cotton stems at 5 (h), 10 (i), and 15 (j) days post-inoculation (h–j). The connecting lines show duplicated sequences in FOV7.

We performed whole-genome sequencing and de novo assembly of FOV7 using single-molecule real-time sequencing technology (PacBio Sequel). A total of 738,287 raw reads with a combined size of 8.07 gigabases (Gb) were obtained, resulting in a sequencing depth of approximately 124×. The initial assembly was performed using MECAT2 [28], and the PacBio assembly was polished using Illumina paired-end data. This yielded a preliminary assembly consisting of 54 contigs, with an N50 size of 3.56 megabases (Mb) (Table 1). To obtain chromosome-level scaffolds, the assembly was aided by high-throughput chromosome conformation capture (Hi-C) data (Figure S1). Ultimately, a genome sequence of 64.16 Mb was obtained, consisting of 30 scaffolds (Table 1). The largest 16 scaffolds (>1 Mb) accounted for 97.27% of the total genome sequence and are displayed by circus plot (Figure 1). The scaffold N50 was reached at 5.08 Mb. The integrity of the genome was evaluated using BUSCO (version 5.1.3) [43], with a completeness score of 99.2%. By combining PacBio, Illumina, and Hi-C data, we achieved an almost chromosome-level genome of FOV7 (Table 1) (referred to as “almost” due to the lack of evidence regarding the exact number of chromosomes in FOV7).

The genome annotation was performed using the Funannotate pipeline. To improve transcript prediction, we collected spores of FOV7 and FOV7-infected cotton stems at 5, 10, and 15 days post-inoculation (dpi) for transcriptome sequencing. The data for 5 dpi and 10 dpi were released in our previous study [41]. The transcriptome sequencing reads were aligned to the FOV7 genome, showing average alignment rates of 98.19%, 0.08%, 0.69%, and 2.84% for the respective time points. A correlation was observed between the progression of pathogen infection and the accumulation of pathogens in the cotton stems (Figure S2). Combining the transcriptome data, a total of 19,633 protein-coding genes were predicted (Figure 1), with 1265 (6.44%) predicted as secreted proteins and 495 classified as effectors (Table S3). Heatmap analysis revealed that the expression of most effector genes is induced in *planta* (Figure S3). Repeat sequences accounted for 18.42% of the total genome size in FOV7, with retroelements comprising 5.43% and DNA transposons comprising 7.28%. Notably, the scaffold regions sca04, sca08, sca12, sca14, sca15, and sca16 exhibited a high density of DNA transposons, with segmental duplications present on these scaffolds (Figure 1).

### 3.2. Phylogenomic Analysis Revealed the Divergence or Closeness in the Evolutionary Relationships among Different Races of Fov

**Figure 2:**
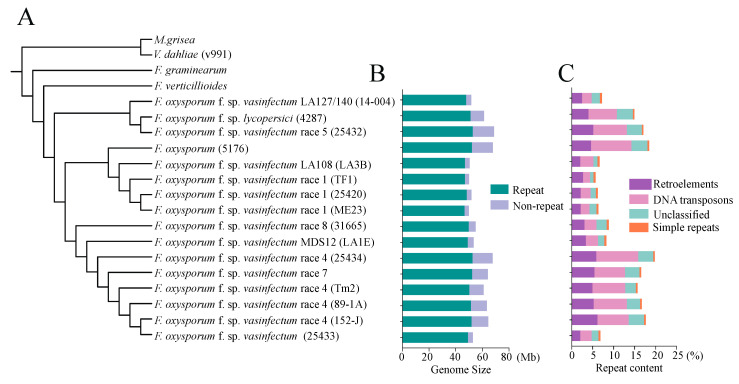
Phylogenomic relationship and genome features of sequenced *F. oxysporum* f. sp. *vasinfectum* isolates. (**A**) Phylogenetic tree of *Fov* species based on all homologous genes. (**B**) Genome size including repeat and non-repeat sequences. (**C**) Percentage of different categories of repeats in the FOV7 genome.

In order to investigate the phylogenetic relationship among different *Fov* races at the whole-genome level, we selected 12 *Fov* isolates with completed genome sequencing, along with other fungi species and FOV7 isolates, to identify orthologs and study their phylogenetic associations. Our phylogenetic analysis results revealed that the twelve *Fov* isolates could be divided into four distinct subgroups (Figure 2A). Specifically, FOV4, FOV7 25433, FOV8, and MDS12 were found to belong to the same subgroup. On the other hand, FOV1, LA108, and *F. oxysporum* 5176 comprised a separate subgroup. Another subgroup consisted of FOV5, LA127/140, and *Fol* 4287 (Figure 2A). The results demonstrate notable divergence or closeness in the evolutionary relationships among different races/strains of *Fov*. Notably, phylogenomic analysis indicated that FOV7 and FOV4 isolates exhibit the closest relationship (Figure 2A). Moreover, FOV7 and FOV4 isolates exhibit significant similarities in terms of genome size and the percentage of repeat sequences, including retroelements and DNA transposons (Figure 2B,C). Among the 12 sequenced isolates, the isolates of FOV4, FOV5, and FOV7 exhibit the largest genome sizes, exceeding 60 Mb. The lengths of total non-repeat sequences are similar among different races or isolates, while the lengths of total repeat sequences vary significantly (Figure 2B). The proportions of repeat sequences in these three race isolates exceed 17%, with transposable elements exceeding 15%. In contrast, the proportions of repeat sequences in other isolates range from 6.25% to 9.52% and transposable elements range from 5.16% to 8.34% (Figure 2A,B). These results imply that the large genome size of FOV4, FOV5, and FOV7 is primarily due to a higher proportion of repeat sequences, particularly the amplification of transposable elements (TEs). 

### 3.3. Comparative Genome Analysis Identified Lineage-Specific Regions of FOV7

**Figure 3:**
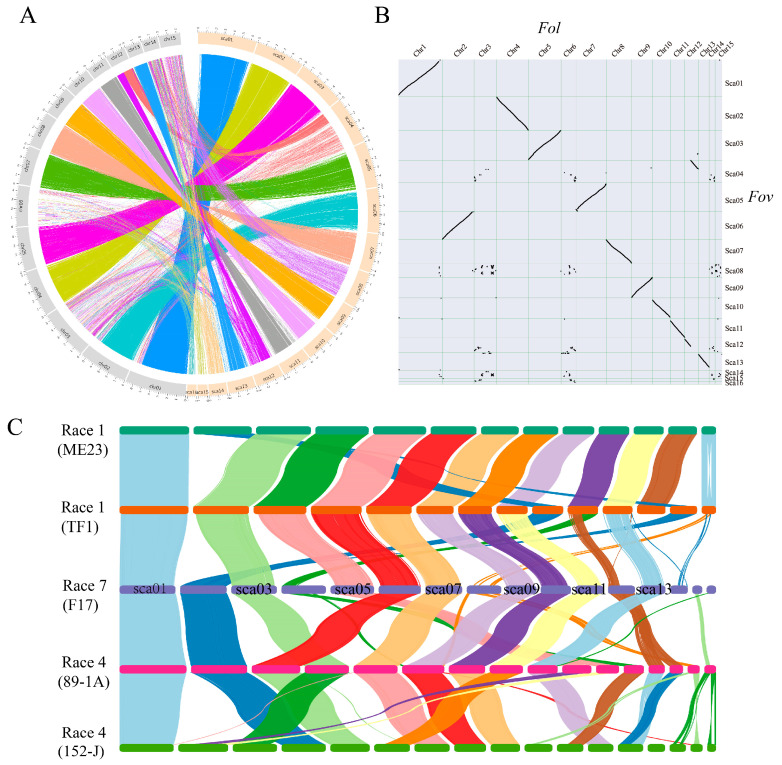
Comparative genome analysis between *Fov* race 7 and *Fol*, and genome synteny assessment among diverse *Fov* isolates. (**A**) Genome alignment of FOV7 and *Fol* strain 4287. (**B**) Co-linearity analysis of gene pairs between FOV7 and *Fol*. (**C**) Genome alignment depicting syntenic regions among diverse *Fov* isolates. Lines between chromosomes (Chr) and each scaffold (Sca) represent syntenic regions.

To unveil the genomic features that potentially contribute to the speciation and pathogenicity of *Fov*, we performed a whole-genome synteny comparison between FOV7 and *Fol* 4287. The analysis revealed that 10 scaffolds of FOV7 exhibited collinearity with the conserved core genome of *Fol*, while the other 6 scaffolds displayed low similarity to the lineage-specific (LS) genome of *Fol* (Figure 3A,B). Therefore, these 10 scaffolds are considered the core scaffolds of FOV7, representing 71.06% of the total genome size. On the other hand, sca04, sca08, sca12, sca14, sca15, and sca16, referred to as LS scaffolds, comprise 26.20% of the total genome size. In contrast, the LS scaffolds of FOV7 predominantly contain transposable elements (TEs), with a TE proportion of 58.45% and DNA transposons of 66.19%. This proportion is similar to *Fol*, but there are some differences, as the proportion of TEs and DNA transposons in the LS region of FOV7 is lower than that in *Fol* (TEs: 74%, DNA transposons: 95%) [20]. Additionally, we noticed that scaffolds 4 and 12 in FOV7 contain both LS and core sequences, with the core sequences showing synteny with the core chromosome 12 in *Fol* 4287 (Figure 3A,B). This pattern is also evident in the collinearity comparison between FOV4 isolate 152-J and *Fol* 4287 (Figure S4A). However, the scaffolds in FOV1 (isolate ME23 and TF1) exhibiting synteny with the core chromosome 12 in *Fol* 4287 lack LS sequences (Figure S4B). 

Further synteny comparison between different *Fov* races was conducted. Genomic collinearity analysis revealed a high sequence similarity between FOV7 isolates and those belonging to FOV1 and FOV4, which implicates the high-quality assembly of the FOV7 genome (Figure 3C). Additionally, we observed that the lineage-specific scaffolds of FOV7 exhibit lower collinearity with FOV1 and FOV4. However, the collinearity between FOV7 and FOV4 in this region is higher than that between FOV7 and FOV1, particularly on scaffold 12 (Figure 3C), which is consistent with their evolutionary relationship. The high collinearity between FOV4 and FOV7 in the core region partly explains why previous studies, using vegetative compatibility analysis and limited DNA markers, were unable to identify genetic differences between these two races (VCG 0114) [13,14]. The specific DNA sequence of LS regions can serve as potential diagnostic markers to effectively distinguish *Fov* races. Additionally, a unique *Tfo1* transposon insertion in the phosphatase permease gene (PHO) was discovered in a race 4 isolate collected in California (designated as Cal race 4), but this insertion was not found in FOV7 isolate 25433 [44]. However, our findings demonstrated that the *Tfo1* transposon insertion was also present in the race 7 isolate F17 of this study. These results indicate that FOV7 can be divided into two genotypes: N type (absence of *Tfo1* transposon insertion in the PHO gene) and T type (presence of he *Tfo1* transposon insertion in the PHO gene). Furthermore, we observed that the FOV7 strain used in this study and strain 25433, despite their close evolutionary relationship, exhibit significant differences in genome size and TE content (Figure 2). This suggests that FOV7 has undergone evolution and differentiation in China, with TEs playing a crucial role. 

### 3.4. Identification of Potential Effector Genes

Secreted proteins play a crucial role during the infection process of plant pathogens. In total, 1265 proteins exhibiting classical characteristics of secreted proteins were identified, accounting for 6.44% of the total predicted proteome. Among these, 495 were predicted as effectors (Table S3). Heatmap analysis revealed that the expression of most effector genes is induced in *planta* (Figure S3). In *Fol*, 14 SIX (secreted in xylem) proteins are identified and 4 of them have been confirmed as genuine effector factors [15,16,17]. Blast analysis founded that there are three genes that share homolog with *SIX9*, and other *SIX* homologous gene were not found in FOV7. To identify potential effectors in FOV7, xylem sap was collected from cotton plants 10 dpi with FOV7, as well as from control plants treated with water. Each sample was replicated three times. Label-free quantitative proteomics was employed to detect proteins secreted in the xylem sap of cotton. We considered only the proteins identified in the infected group as SIX proteins of FOV7. Through this analysis, we identified a total of 19 SIX proteins in FOV7, which were named *Fov*SIX1-*Fov*SIX19 (Table 2). The majority of these *Fov*SIX contain signal peptides and are predicted to be effectors. Furthermore, we observed that the expression of most of these *FovSIX* genes is induced in *planta* (Table 2), indicating their potential role in mediating host–pathogen interactions. Among these nineteen *FovSIX* genes, one of three homologs of *SIX9* was found (*Fov7_sca04_1092*, *FovSIX6*). However, through inoculation experiments, we discovered that the knockout of this gene did not affect the pathogenicity of FOV7 on cotton plants (Figure S5). These *Fov*SIX proteins provide a genetic resource for the identification of genuine effectors in FOV7. 

### 3.5. FovSIX16 Was Required for the Full Virulence of FOV7

**Figure 4:**
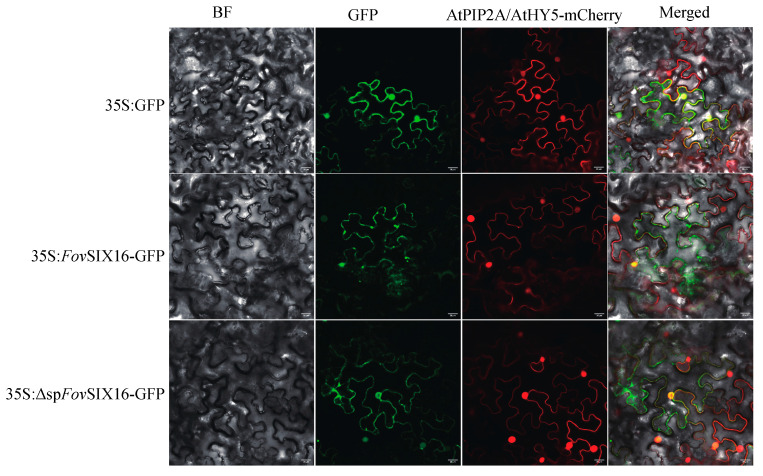
Subcellular location of *Fov*SIX16/*Fov*ΔspSIX16-GFP fusion proteins in *Nicotiana benthamiana* leaves. GFP, *Fov*SIX16-GFP, and *Fov*ΔspSIX16-GFP were co-infiltrated with AtPIP2A-mCherry (a plasma membrane intrinsic protein) and AtHY5-mCherry (a nucleus-located protein), respectively. GFP was used as the control. The term “*Fov*SIX16” represents a full-length sequence, while the term “*Fov*ΔspSIX16” represents *Fov*SIX16 with signal peptide sequence deletion. Scale bars indicate 20 μm.

Among these *Fov*SIX proteins, Fov7_sca15_0202 was identified as a putative effector with eight cysteine residues, and its expression was significantly induced in *planta* (Table 2). Moreover, *Fov*SIX16 was observed to localize in both the plasma membrane and the cell nucleus of tobacco cells (Figure 4). 

**Figure 5:**
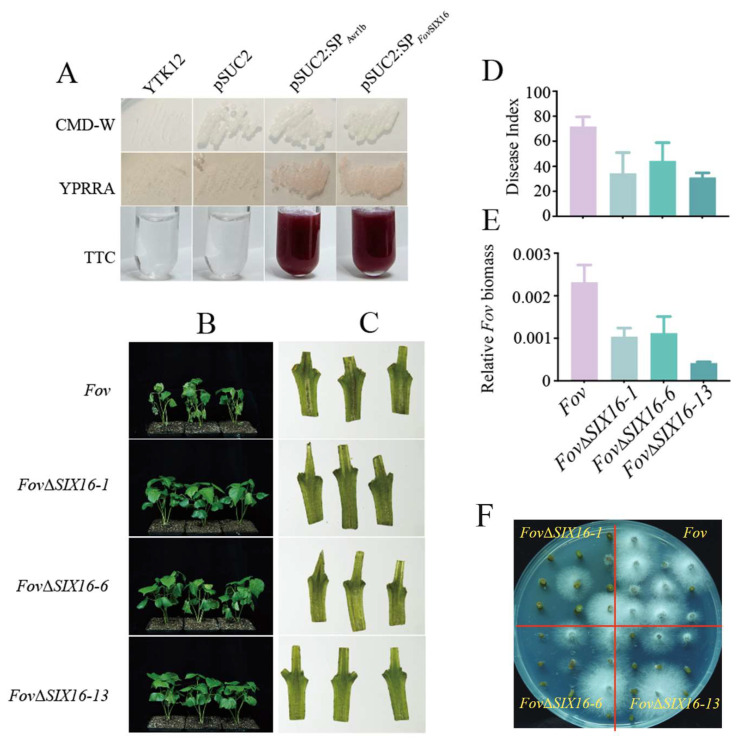
*Fov*SIX16 is a secreted protein playing a crucial role in the virulence of FOV7 on cotton plants. (**A**) The secretion of *Fov*SIX16 was assessed using a yeast signal-sequence trap system assay. Plasmid contained the signal peptide sequence of *Fov*SIX16 (SP*_Fov_*_SIX16_); Avr1b (SP_Avr1b_) was under the control of the pSUC2 promoter and transformed into a YTK12 yeast strain. Yeast expressing the empty pSUC2 vector served as a negative control and yeast expressing pSUC2-SP_Avr1b_ acted as the positive control. (**B**) Disease symptoms of cotton plants at 20 days post-inoculation with FOV7 and *Fov*Δ*SIX16* knockout mutants. (**C**) Vascular bundle discoloration in longitudinal sections of cotton stems at 20 days post-inoculation with FOV7 and *Fov*Δ*SIX16* knockout mutants. (**D**) Disease index (DI) statistics at 3 weeks after *Fov* inoculation. (**E**) Quantification of fungal biomass by measuring the relative content of *Fov* DNA in the stem of cotton plants. (**F**) Fungal recovery assay involved incubating short sections cut from inoculated plants on potato dextrose agar (PDA) medium.

To confirm the secretion activity of *Fov*SIX16, we employed the yeast signal-sequence trap system. The results demonstrated that yeast transformants expressing the signal peptide of *Fov*SIX16 displayed a pink color, consistent with the positive control transformants expressing the Avr1b signal peptide, while the negative control remained colorless (Figure 5A). This indicates the secretion activity of *Fov*SIX16. To investigate the impact of *FovSIX16* on FOV7 virulence, we performed homologous recombination to generate *Fov*Δ*SIX16* knockout mutants. Three transformants (*Fov*Δ*SIX16-1*, *Fov*Δ*SIX16-6*, and *Fov*Δ*SIX16-13*) were selected for further experiments. Compared to the wild-type strain, the *Fov*Δ*SIX16* knockout mutant strains did not significantly affect hyphal growth rate (Figure S6A). The sporulation quantity only showed a slight alteration in *Fov*Δ*SIX16-6*, while the other two transformants had no notable change (Figure S6B). Furthermore, we inoculated *glr4.8* (with a resistance gene *GhGLR4.8* knockout) [41] with both the wild-type strain and the *Fov*Δ*SIX16* knockout mutants, and found that the deletion of *FovSIX16* led to a significant decrease in FOV7 virulence. The *glr4.8* cotton plants inoculated with the *Fov*Δ*SIX16* mutants exhibited significantly milder symptoms and reduced brown coloration of the vascular tissue compared to those inoculated with the wild-type strain (Figure 5B,C). Statistical analysis showed that the disease index (DI) of *glr4.8* cotton plants inoculated with the *Fov*Δ*SIX16* mutants was significantly lower compared to that of the wild-type strain (Figure 5D), and this was accompanied by a reduced fungal biomass (Figure 5E). Additionally, the recovery assay was performed using the inoculated cotton stems, and the results revealed a more vigorous hyphal growth around the stems of *glr4.8* mutant plants when inoculated with the wild-type strain, as compared to the *Fov*Δ*SIX16* mutants (Figure 5F). These results strongly support the significant role of *FovSIX16* in FOV7 virulence and its necessity for full virulence.

## 4. Discussion

Currently, a total of 30 released *Fov* genomes have been documented, excluding the FOV7 genome [23,24,26,27]. Out of these, 12 genomes were sequenced using third-generation sequencing technologies. Among them, five isolates were sequenced using PacBio sequencing (one each for races 1 and 4, with three of indeterminate race), while seven isolates were sequenced using Oxford Nanopore sequencing (two belonging to race 1, one to race 5, three to race 4, and one to race 8). The remaining 18 genomes were sequenced using Illumina second-generation sequencing, encompassing one FOV7 isolate, labeled NRRL 25433. However, there have been no reports of long-read genome sequencing for FOV7 until now. In this study, we utilized a combination of PacBio and Hi-C sequencing to obtain a high-quality genome of FOV7 (Table 1, Figure 1). This fills the gap in the availability of a highly contiguous FOV7 genome and provides a valuable addition to the *Fov* genome database. Compared to second-generation sequencing, third-generation sequencing offers longer read lengths, resulting in higher continuity of the *Fov* genome assembly. Based on the released data [23,26,27], the contig/scaffold count of assembled *Fov* using third-generation sequencing ranges from 13 to 86, with an N50 reached from 3.33 Mb to 4.89 Mb. In contrast, the assembly of the *Fov* genome using second-generation sequencing yields a higher number of contigs, reaching several thousand. With the advancement of sequencing technologies, obtaining high-quality *Fov* genomes has become more feasible, providing a solid foundation for studying the evolution and pathogenic mechanisms of *Fov*. Using Hi-C assisted assembly, we achieved near-chromosomal level assembly of the FOV7 genome, with the scaffold N50 reached at 5.08 Mb (Table 1). However, the number of chromosomes in FOV7 still remain unknown. The FOV1 isolate TF1 was assembled into only 17 contigs [23], while the FOV1 isolate ME23 was assembled into 13 contigs, and FOV4 isolates Tm2 and 152-J were assembled into 16 and 18 contigs, respectively [26,27]. In comparison, *Fol* 4287 has been reported to have 15 chromosomes [20]. The FOV7, FOV4, and *Fol* 4287 genomes exhibit similar sizes, and our analysis reveals a notable collinearity between FOV7 and both *Fol* 4287 and FOV4 genomes (Figure 2 and Figure 3). Based on these results, it is hypothesized that different *Fov* races may possess varying numbers of chromosomes, with FOV4 and FOV 7 potentially having nearly the same chromosome count as *Fol* 4287. However, to confirm this hypothesis, karyotype analysis of fungi using cytological or molecular biology methods such as traditional microscopy, scanning electron microscopy, the germ tube burst method, and pulse field gel electrophoresis is necessary. Additionally, both FOV4 and FOV7 possess two scaffolds containing both LS and core sequences, with the core sequences showing synteny with core chromosome 12 in *Fol* 4287. However, this patten is absent in the collinearity comparison between FOV1 (isolates ME23 and TF1) and *Fol* 4287 (Figure S4). Furthermore, there is a higher content of transposable elements (TEs) and larger genome sizes in race 7 and race 4 when compared to race 1 (Figure 2). These findings suggest that the increased transposable element content during the evolution of race 7 and race 4 likely contributed to genome expansion and the subsequent rearrangement of genome sequences.

*Fov* isolates have traditionally been classified into eight pathogenic races (races 1–8) based on their varying ability to infect different plant species, including soybean, tobacco, okra, lupine, and alfalfa, as well as certain *Gossypium* spp. and cultivars [6,46]. However, with the collection of more isolates and advances in research, the previous race designations for *Fov* have been found to be both invalid and impractical [13,47]. Consequently, new isolates are now characterized by DNA sequencing and vegetative compatibility group (VCG) analyses to determine biotypes, genotypes, and genetic variations among races. The development of molecular DNA technology has greatly facilitated the identification of genotypes. Multigene sequence analyses, particularly involving housekeeping genes, have enabled the subdivision of *Fov* races into four lineages [7]. Evolutionary analysis based on orthologous genes from the entire genome has revealed that the 13 sequenced *Fov* isolates can be categorized into 3 subgroups: subgroup 1 (including races 5 and the LA127/140 genotype), subgroup 2 (including race 1 and the LA108 genotype), and subgroup 3 (including race 4, 7, and 8 and the MDS12 genotype) (Figure 2). As more *Fov* isolates are sequenced, our comprehensive knowledge of *Fov* will continue to expand. The utilization of whole-pathogen genome sequencing has further enhanced our understanding of the evolutionary relationships among different *Fov* races.

There are more than 21 vegetative compatibility groups (VCGs) and 2 distinct pathotypes that have been described for *Fov*. This includes six of the recognized races (1, 2, 3, 4, 6, and 8) of *Fov*, as well as two Australian biotypes [10]. While traditional methods have not successfully identified genetic differences between FOV4 and FOV7, our comparative genomic analysis revealed significant differences in lineage-specific regions between these two races, despite their high genomic similarity (Figure 3C). On the other hand, minimal differences were observed among different isolates of FOV4 (Figure 3C). These findings suggest that FOV4 and FOV7, although belonging to the same VCG, are likely to be different races. Previous studies showed that race 4 is more pathogenic to Pima cotton, while race 7 is more aggressive toward Acala cotton (*G. hirsutum* L.) [8]. In order to better differentiate different races, it is necessary to conduct comprehensive pathogenicity tests with an expanded host range. The integration of whole-genome analysis, VCG detection, and pathogenicity testing will provide powerful tools for categorizing different races of *Fov*.

Analysis of the xylem sap proteome of *Fol*-infected tomato plants led to the identification of a subgroup of secreted proteins known as the secreted in xylem (Six) 1–14 [15,16,17]. Homologs of SIX genes from other *formae speciales* of *F. oxysporum* were also identified. In *Fusarium oxysporum* f. sp. *cubense* (*Foc*), seven SIX homolog genes (*SIX1*, *SIX2*, *SIX6*, *SIX7*, *SIX8*, *SIX9*, *SIX10*, and *SIX13*) were discovered [48]. The SIX6 homologous gene was exclusively found in Australian *Fov* isolates, distinguishing them from non-Australian isolates that lack this gene [49]. We found that *SIX9* was the only *SIX* gene with a homolog in FOV7, with three genes identified as *Fov7_sca04_1103*, *Fov7_sca16_0086* and *Fov7_sca04_1092*. These three genes, similar to *Fol*, were found to be located in the LS region. Interestingly, variation in the sequence of the *SIX9* homologous gene was observed among different *Fov* races or isolates. LA108, LA127, and MDS12 did not possess the *SIX9* homologous gene, whereas FOV4 and FOV7 exhibited the SIX9 homologous gene. Therefore, the SIX9 homologous gene in *Fov* can serve as a preliminary marker for differentiating pathotypes or isolates. 

Additionally, following the identification of SIX proteins in *Fol*, we carried out SIX protein identification in FOV7. By analyzing the xylem sap proteome of FOV7-infected cotton plants, we identified 19 *Fov*SIX proteins (Table 2). Unlike *Fol*, where all *Fov*SIX proteins are found in the LS region, only 10 out of the 19 *Fov*SIX proteins in FOV7 were located in the LS region (Table 2). This difference in distribution may be attributed to the distinct properties exhibited by SIX proteins among different specialized pathogens. Among the 19 identified *Fov*SIX proteins, we observed the presence of the SIX9 homolog protein, Fov7_sca04_1103 (Table 2). However, the knockout of this gene did not impact the pathogenicity of FOV7 on cotton (Figure S5), suggesting that SIX proteins may have diverse functions among specialized strains. Another possible reason could be the functional redundancy of SIX9 in FOV7. Notably, *FovSIX16* emerged as an essential contributor to FOV7 pathogenicity, and we confirmed its secretion functionality (Figure 5). *FovSIX16* encodes PEP1, and in previous studies, Chakrabarti et al. identified two potential effector genes, PEP1 and PEP2, by analyzing *Fov* genes expressed during cotton infection [49]. We observed that *FovSIX16* exhibits 96% DNA similarity with PEP1 and shares the same sequence as the one found in FOV4, suggesting a closer resemblance between FOV7 and FOV4 in terms of pathogenicity compared to other pathotypes. However, this gene is absent in FOV1, LA108, LA127/LA140, and the MDS12 genotype. 

In conclusion, the high-quality FOV7 genome enriched the *F. oxysporum* genome repository, enabling the exploration of pathogenic factors and the development of diagnostic markers through comparative genomics. The identification of the FovSIX protein and the potential pathogenic factor FovSIX9 provides valuable insights into the mechanisms underlying Fov’s pathogenicity and for unraveling effectors in other *F. oxysporum* species.

## Figures and Tables

**Table 1:** Summary of de novo genome assembly and annotation of *Fusarium oxysporum* f. sp. *vasinfectum* race 7.

Genomic Feature	*Fov* Race 7
Total length of contigs	64,259,633
Total length of assemblies	64,156,821
Percentage of anchoring	98.60%
Number of contigs ^a^	54
Contig N50 (bp)	3,564,359
Number of scaffolds ^b^	30
Scaffold N50 (bp)	5,083,699
GC content	47.64%
Percentage of repeat sequences	18.42%
Number of genes	19,633
Number of transcripts	20,417
BUSCO completeness	99.20%

^a^ PacBio CLR+ Illumina-corrected contigs; ^b^ Hi-C-assembled genome sequences.

**Table 2:** Features of the SIX proteins identified in FOV7-infected cotton xylem saps.

SIX ID	Gene ID	AA Length	SP ^a^	Induced in *planta*	Effector Prediction ^b^	SIX Homologs	Core/LS Scaffold
FovSIX1	Fov7_sca01_1094	212	Yes	No	No	ND	Core
FovSIX2	Fov7_sca02_1180	567	Yes	Yes	No	ND	Core
FovSIX3	Fov7_sca04_0306	275	Yes	Yes	Yes	ND	LS
FovSIX4	Fov7_sca04_0935	127	Yes	No	No	ND	LS
FovSIX5	Fov7_sca04_1062	107	Yes	Yes	Yes	ND	LS
FovSIX6	Fov7_sca04_1092	118	Yes	Yes	Yes	SIX9	LS
FovSIX7	Fov7_sca04_1108	87	Yes	No	Yes	ND	LS
FovSIX8	Fov7_sca05_0196	432	Yes	Yes	No	ND	Core
FovSIX9	Fov7_sca05_0374	290	Yes	Yes	No	ND	Core
FovSIX10	Fov7_sca05_1077	106	No	No	Yes	ND	Core
FovSIX11	Fov7_sca06_0097	274	Yes	No	Yes	ND	Core
FovSIX12	Fov7_sca07_0495	154	No	Yes	Yes	ND	Core
FovSIX13	Fov7_sca07_1331	379	Yes	Yes	NO	ND	Core
FovSIX14	Fov7_sca10_0956	419	Yes	Yes	NO	ND	Core
FovSIX15	Fov7_sca15_0057	102	Yes	Yes	Yes	ND	LS
FovSIX16	Fov7_sca15_0202	269	Yes	Yes	Yes	ND	LS
FovSIX17	Fov7_sca15_0203	107	Yes	Yes	Yes	ND	LS
FovSIX18	Fov7_sca16_0047	130	Yes	Yes	Yes	ND	LS
FovSIX19	Fov7_sca18_0157	148	Yes	Yes	Yes	ND	LS

^a^ signalP 5.0 was used to predict whether the *Fov*SIX proteins have a signal peptide (SP) sequence. ^b^ EffectorP 3.0 [45] was used to predict whether the *Fov*SIX proteins act as possible effectors. “ND” indicates the absence of any SIX homologs detected.

## Data Availability

The genome data presented in this report are available through GenBank under BioProject accession number PRJNA1073426 and BioSample accession number SAMN40479352.

## Supplementary Materials

The following supporting information can be downloaded at: https://www.mdpi.com/article/10.3390/jof10040242/s1, Table S1. *Fov* isolates used in this study; Table S2: Primers used in this study; Table S3: Summary of predicted secreted proteins and effectors in FOV7; Figure S1: Hi-C heatmap showing the chromatin-interaction density among scaffolds of the FOV7 genome; Figure S2: The alignment rate of RNA-seq reads mapping to FOV7 genome; Figure S3: Heatmap showing the expression pattern of the predicted effector genes in vitro and in *planta* at 5 dpi, 10 dpi, and 15 dpi; Figure S4. Genome synteny assessment between *Fov* isolates and *Fol* 4287; Figure S5: The homologous gene of *SIX9* in FOV7 is not necessary for the complete virulence of FOV7; Figure S6: Impacts of *FovSIX16* knockout on hyphal growth rate and sporulation quantity.
