# Supplementary material for: Characterization of the High-Quality Genome Sequence and Virulence Factors of Fusarium oxysporum f. sp. vasinfectum Race 7
Source: J Fungi (Basel). 2024 Mar 23;10(4):242. doi: 10.3390/jof10040242 (PMC11051352; doi:10.3390/jof10040242)
Supplement: Supplementary file 1 [file jof-10-00242-s001.zip › Supplementary Figure S1-S6.pdf]

Characterization of the high-quality genome sequence and virulence factors of *Fusarium oxysporum* f. sp. *vasinfectum* race 7

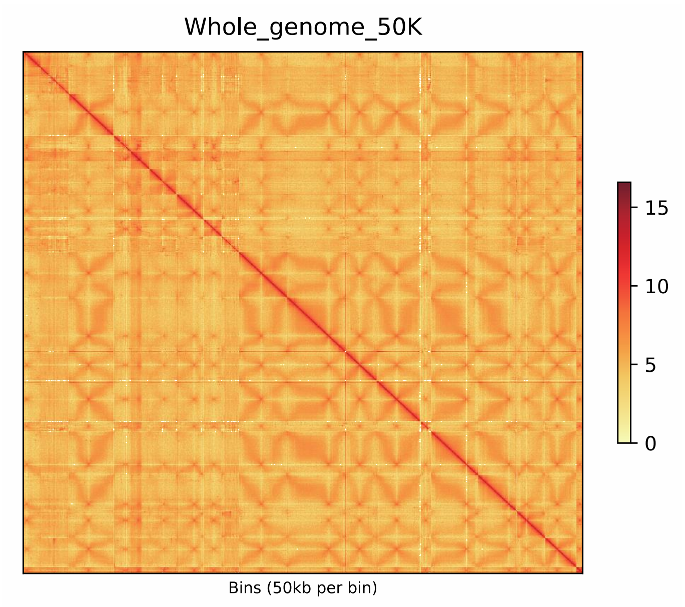

Supplementary Figure S1. Hi-C heatmap showing the chromatin interaction density among scaffolds of FOV7 genome. The Hi-C heatmaps are shown at 50 kb resolution.

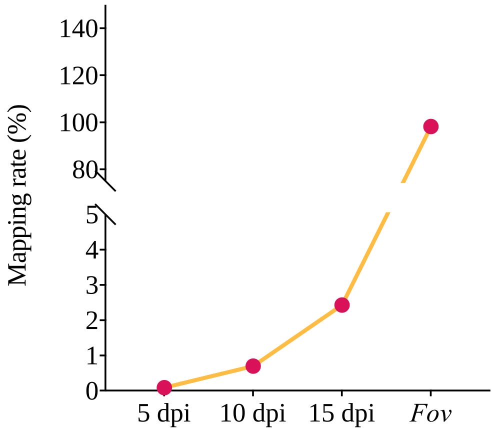

Supplementary Figure S2. The alignment rate of RNA-seq reads mapping to FOV7 genome.

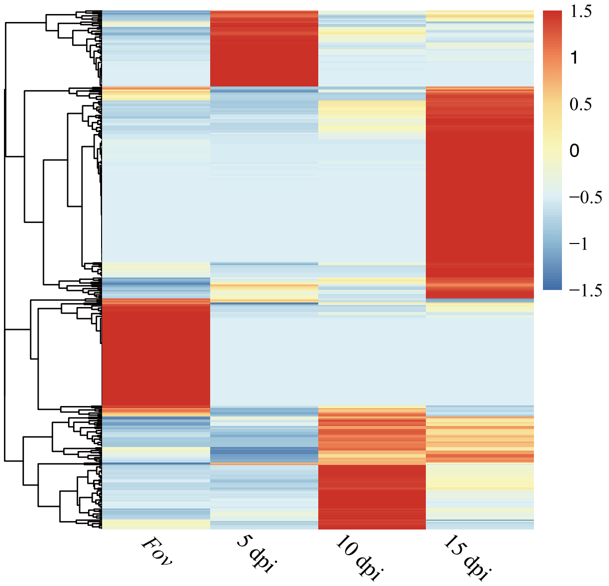

Supplementary Figure S3. Heatmap show the expression pattern of the predicted effector genes *in vitro* and *in planta* at 5 dpi, 10 dpi and 15 dpi.

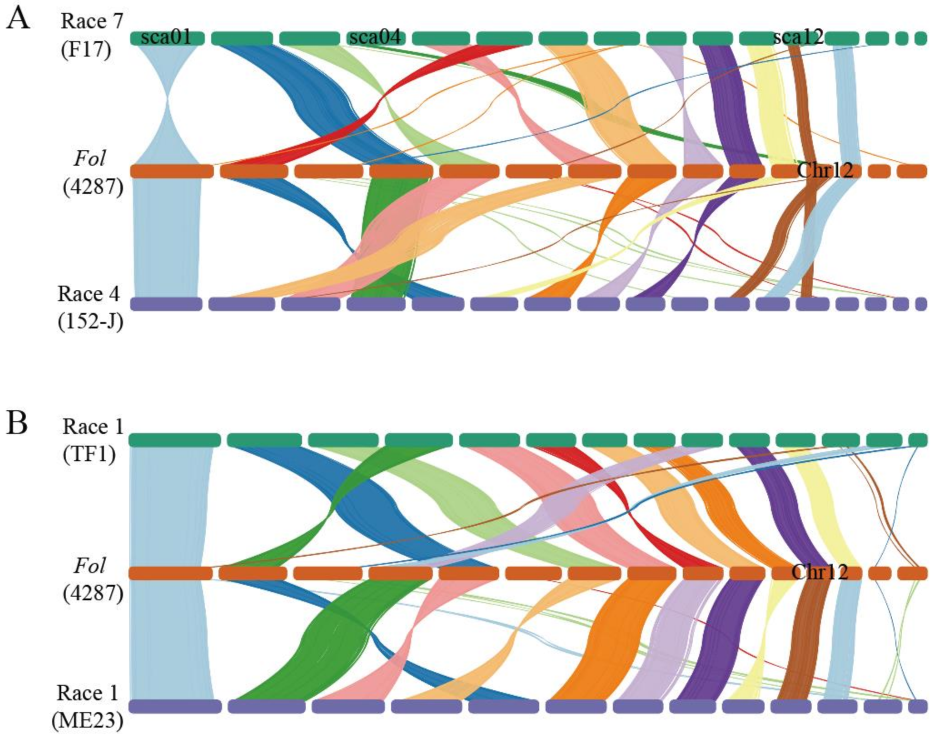

Supplementary Figure S4. Genome synteny assessment between *Fov* isolates and *Fol* 4287. A, genome synteny comparison between isolates of FOV4, FOV7 and *Fol* 4287. B, genome synteny comparison between isolates of FOV1 and *Fol* 4287.

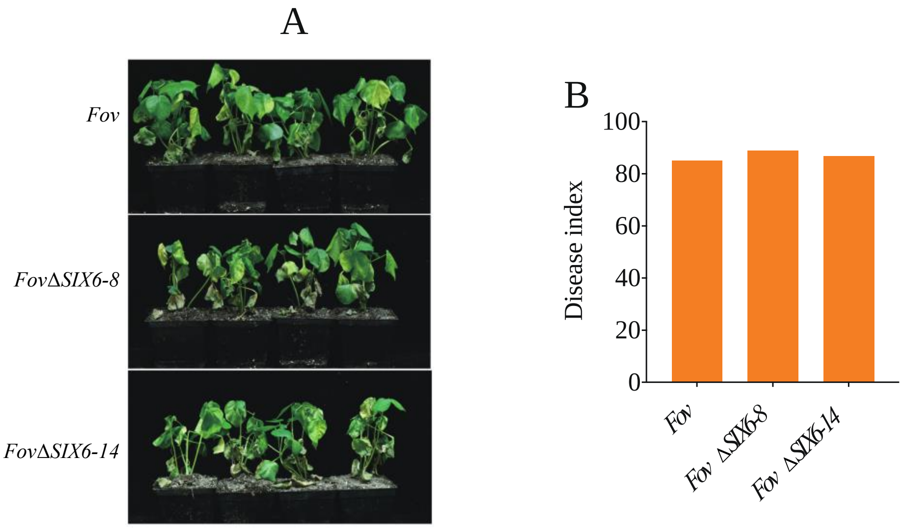

Supplementary Figure S5. The homologous gene of *SIX9* in FOV7 is not necessary for the complete virulence of FOV7. **A**, Disease symptoms of cotton plants at 20 days post-inoculation with FOV7 and *Fov*ΔSIX6 knockout mutants. **B**, Disease index (DI) statistics at 3 weeks after *Fov* inoculation.

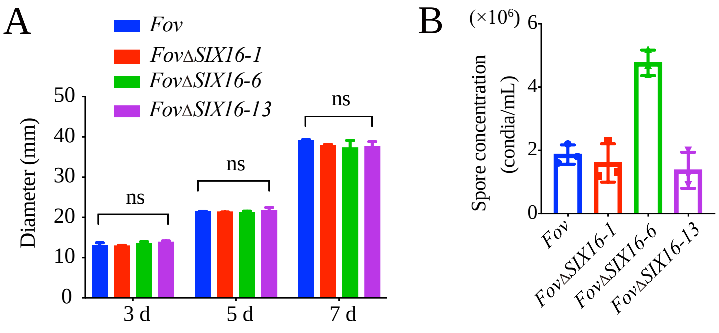

Supplementary Figure S6. Impacts of *FovSIX16* knockout on hyphal growth rate and sporulation quantity. **A,** Comparison of hyphal growth rate between wild type strain and *Fov*Δ*SIX16* knockout mutants. **B**, Comparison of sporulation quantity between wild type strain and *Fov*Δ*SIX16* knockout mutants.
